# Multidisciplinary consensus statement on the clinical management of patients with pancreatic cancer

**DOI:** 10.1007/s12094-020-02350-6

**Published:** 2020-04-21

**Authors:** E. Martin-Perez, J. E. Domínguez-Muñoz, F. Botella-Romero, L. Cerezo, F. Matute Teresa, T. Serrano, R. Vera

**Affiliations:** 1 Department of Surgery, Hospital Universitario de La Princesa, Diego de Leon 62, 28006 Madrid, Spain; 2 Department of Gastroenterology and Hepatology, Hospital Clínico Universitario de Santiago de Compostela, Santiago de Compostela, Spain; 3 Department of Endocrinology, Hospital General Universitario, Albacete, Spain; 4 Department of Radiation Oncology, Hospital Universitario de La Princesa, Madrid, Spain; 5 Department of Radiology, Hospital Clínico San Carlos, Madrid, Spain; 6 Department of Pathology, Hospital Universitario de Bellvitge, L’Hospitalet de Llobregat, Barcelona, Spain; 7 Oncology Program, CIBEREHD National Biomedical Research Institute on Liver and Gastrointestinal Diseases, Instituto de Salud Carlos III, Madrid, Spain; 8 Department of Medical Oncology, Complejo Hospitalario de Navarra, Pamplona, Spain

**Keywords:** Pancreatic cancer, Diagnosis, Surgery, Chemotherapy, Radiotherapy, Guidelines

## Abstract

Pancreatic cancer (PC) remains one of the most aggressive tumors with an increasing incidence rate and reduced survival. Although surgical resection is the only potentially curative treatment for PC, only 15–20% of patients are resectable at diagnosis. To select the most appropriate treatment and thus improve outcomes, the diagnostic and therapeutic strategy for each patient with PC should be discussed within a multidisciplinary expert team. Clinical decision-making should be evidence-based, considering the staging of the tumor, the performance status and preferences of the patient. The aim of this guideline is to provide practical and evidence-based recommendations for the management of PC.

**Electronic supplementary material:**

The online version of this article (10.1007/s12094-020-02350-6) contains supplementary material, which is available to authorized users.

## Introduction

Pancreatic cancer (PC) represents the fourth leading cause of cancer-related death, and it has been estimated to become the second by 2030 [1, 2]. PC is divided into four general categories: resectable, borderline resectable, locally advanced/unresectable, and metastatic. Surgical resection remains the primary curative treatment for patients with PC, although only 15–20% will present with initially resectable disease. Approximately 30–40% of patients show locally advanced PC, and another 40% have distant metastatic disease [3–5].

Over the past decades, there has been considerable improvement in imaging and surgical techniques, and more effective chemotherapy and radiotherapy techniques have been developed [6, 7]. Decisions about the appropriate diagnostic and therapeutic strategy for each patient with PC should involve a multidisciplinary team involving radiologists, gastroenterologists, surgeons, medical oncologists, radiation oncologists, endocrinologists, and pathologists with expertise in the management of pancreatic cancer [8]. The aim of this guideline is to summarize the current evidence and to give practical and evidence-based recommendations for the diagnosis and treatment of PC.

## Methodology

A group of seven experts—one from each Society- from the Spanish Society of Medical Oncology (SEOM), the Spanish Association of Surgeons (AEC), the Spanish Society of Radiation Oncology (SEOR), the Spanish Society of Endocrinology (SEEN), the Spanish Society of Digestive Pathology (SEPD), the Spanish Society of Medical Radiology (SERAM), and the Spanish Society of Pathology (SEAP) met to discuss and provide a multidisciplinary consensus on the management of pancreatic cancer. In this consensus, we provide 40 clinical questions addressing diagnosis, chemotherapy, radiation therapy, surgical treatments, and supportive therapy. The available medical literature was reviewed, and answers are given to each clinical question classified by scientific levels of evidence and the strength of recommendation [9].

## Diagnosis and staging

### What is the best imaging modality for the diagnosis and staging of PC?

Multidetector computed tomography (MDCT) with angiography (at the pancreatic arterial [40–50 s] and portal venous [65–70 s] phases) is currently the worldwide imaging modality of choice for the evaluation of PC. MDCT provides three-dimensional (3D) multiplanar reconstruction images that enable the determination of tumor size, extent (vascular involvement), and spread [3, 10, 11] (quality of evidence: A; strength of recommendation: strong).

Magnetic resonance imaging (MRI) may be helpful for differentiating an inflammatory pancreatic mass from pancreatic adenocarcinoma, detecting isoattenuating PC, characterizing small tumors (< 1 cm) or hepatic lesions, or detecting metastases to the liver [12, 13].

### What are the indications for endoscopic ultrasonography (EUS)?

EUS is indicated to diagnose PC in cases of inconclusive MDCT findings, to obtain cytohistological samples for pathological confirmation, and—complementary to MDCT—for loco-regional staging [13–16] (quality of evidence: B; strength of recommendation: moderate).

EUS is particularly useful for the detection of small pancreatic lesions that cannot be identified by other imaging techniques [14]. EUS-guided biopsy is preferred over percutaneous puncture because of its higher diagnostic yield (> 90%), safety and lower risk of seeding [15]. A recent meta-analysis showed that EUS had a sensitivity and specificity of 72% and 90% for T1–T2 staging and 90% and 70% for T3–T4 staging, respectively [16].

### When is cytohistological confirmation necessary before starting treatment?

A pathologic diagnosis is indicated before administration of neoadjuvant therapy in patients with borderline or unresectable lesions, in the presence of metastatic disease or in patients with atypical presentation where a differential diagnosis with other pancreatic masses (autoimmune pancreatitis, lymphoma, chronic pancreatitis, tuberculosis, metastases) is needed. If a biopsy does not confirm malignancy, it should be repeated at least once. A positive biopsy is not required in patients with clinically and radiologically suspected resectable PC before surgical resection because it may result in seeding, interfere with definitive surgery, and delay surgical resection if nondiagnostic [8, 15, 17, 18] (quality of evidence: B; strength of recommendation: strong).

### When is biliary drainage indicated before surgery, and how is it performed?

Early surgery without previous drainage remains the treatment of choice in patients with resectable PC. Preoperative biliary drainage is mainly indicated in patients with cholangitis and in those with obstructive jaundice scheduled for neoadjuvant therapy [19–21]. Endoscopic retrograde placement of a fully covered metal stent is preferred over plastic stents or percutaneous drainage due to a lower complication rate [20, 22]. Endoscopic ultrasound-guided stent placement is an effective and safe alternative [23] (quality of evidence: A; strength of recommendation: strong).

### How is resectability/unresectability of PC defined?

Current criteria for resectability include the absence of distant metastases, no evidence of tumor involvement of major arteries, and, if there is venous invasion, a suitable segment of the superior mesenteric vein below and portal vein above the site of venous involvement to allow for venous reconstruction [8, 10, 12, 24, 25] (Table 1) (quality of evidence: B; strength of recommendation: strong).

**Table 1:** Definition of resectability according to NCCN guidelines [8]

Resectability status	Arterial	Venous
Resectable	No arterial tumor contact: celiac axis (CA), superior mesenteric artery (SMA), or common hepatic artery (CHA)	No tumor contact with the superior mesenteric vein (SMV) or portal vein (PV) or ≤ 180° contact without vein contour irregularity
Borderline resectable	Pancreatic head/uncinate process: Solid tumor with CHA without extension to the celiac axis or hepatic artery bifurcation allowing safe and complete resection and reconstruction Solid tumor contact with the SMA ≤ 180° Solid tumor contact with variant arterial anatomy (e.g., accessory right hepatic artery, replaced right hepatic artery, replaced CHA, and the origin of replaced or accessory artery) and the presence and degree of the tumor should be noted if present, as it may affect surgical planning Pancreatic body/tail: Solid tumor contact with the CA of ≤ 180° Solid tumor contact with the CA of > 180° without involvement of the aorta and with intact and uninvolved gastroduodenal artery (some members prefer these criteria to be in the unresectable category)	Solid tumor contact with the SMV or PV of > 180°, contact of ≤ 180° with contour irregularity of the vein or thrombosis of the vein but with suitable vessels proximal and distal to the site of involvement allowing safe and complete resection and vein reconstruction Solid tumor contact with the inferior vena cava (IVC)
Unresectable	Distant metastases Pancreatic head/uncinate process: Solid tumor contact with SMA > 180° Solid tumor contact with the CA > 180° Solid tumor contact with the first jejunal SMA branch Body and tail: Solid tumor contact with the SMA or CA Solid tumor contact with the CA and aorta	Pancreatic head/uncinate process: Unreconstructible SMV/PV due to tumor involvement or occlusion (can be due to tumor or bland thrombus) Contact with most proximal draining jejunal branch into SMV Body and tail: Unreconstructible SMV/PV due to tumor involvement or occlusion (can be due to the tumor or a bland thrombus)

## Surgical treatment

### When is surgical treatment indicated for PC?

The selection of patients for surgery should be based not only on anatomic criteria (relationship between the tumor and vessels) but also on biological (duration of symptoms and a CA 19–9 level suggestive of localized disease in the absence of jaundice) and conditional factors (a comorbidity profile appropriate for a major abdominal operation and an Eastern Cooperative Oncology Group (ECOG) performance status of 2 or more) [4, 5, 8, 17] (quality of evidence: B; strength of recommendation: strong).

### What operative technique should be used for patients with PC according to the localization?

The only curative treatment for PC is radical surgery. The aim of surgery is to obtain microscopically negative margins (R0). Pancreaticoduodenectomy (Whipple procedure) is the procedure of choice for patients with tumors located in the head of the pancreas and the uncinated process. Patients with tumors of the body or tail of the pancreas are treated with distal pancreatectomy with splenectomy [26] (quality of evidence: A; strength of recommendation: strong).

Although laparoscopic and robotic pancreatectomy is a feasible alternative to open surgery, the evidence regarding clinical and oncologic outcomes is limited [27]. A recent open-label, single-center, randomized controlled trial compared the perioperative outcomes of 66 patients who underwent pancreatoduodenectomy to treat benign, premalignant or malignant conditions, that was performed using a laparoscopic approach or by open-surgery [28]. Patients who underwent a laparoscopic approach showed a significantly shorter length of stay (primary outcome), longer median operative time, and less severe complications (Clavien-Dindo grade > 3 complications), lower Comprehensive Complication Index score and the number of patients with poor quality outcomes, as compared to those who underwent open surgery [28].

### What should be the extent of lymphadenectomy in the surgical treatment of PC?

Pancreatectomy with standard lymphadenectomy including at least 15 lymph nodes should be the procedure of choice in PC. There is no evidence that extended lymphadenectomy results in a survival benefit in PC, and it increases perioperative complications [29, 30] (quality of evidence: A; strength of recommendation: strong).

### When and how is vascular resection performed?

Vascular involvement has traditionally been considered a formal contraindication for resection [5, 8]. Venous resection and reconstruction to achieve R0 resection is an optimal procedure with similar overall survival and morbidity compared to surgery without venous resection [31, 32]. However, arterial resection during pancreatoduodenectomy is associated with increased mortality and morbidity (bowel ischemia, hemorrhage, thrombosis) and is not recommended [33]. Progress in neoadjuvant therapies has allowed the downstaging of tumors with arterial invasion to borderline resectable or resectable disease, making surgical resection more achievable [34]. Despite these advancements, it is currently accepted that arterial reconstruction is only appropriate in highly selected patients in high-volume centers with surgeons who are familiar with the advanced techniques required for reconstruction [35] (quality of evidence: B; strength of recommendation: strong).

### When should a total pancreatectomy be considered in PC?

In patients with PC, a total pancreatectomy should be considered in patients with multifocal PC or locally advanced tumors who undergo pancreatectomy with arterial resection and reconstruction. It may be an alternative to pancreatic anastomosis in highly selected patients with a high-risk pancreas (soft texture and small pancreatic duct) and obese patients with pancreatic fat infiltration [36, 37] (quality of evidence: B; strength of recommendation: weak).

### What is the role of staging laparoscopy for assessing resectability in PC?

Laparoscopy is useful in the discovery of small superficial liver and peritoneal metastases not visible by preoperative MDCT. Staging laparoscopy can be recommended for patients with borderline resectable disease when neoadjuvant treatment is considered and in patients with an increased risk of disseminated disease (tumors in the body and tail of the pancreas, size > 3–4 cm, high CA 19.9 levels, presence of ascites or large regional lymph nodes), or highly symptomatic (back pain, severe weight loss) [8, 38, 39] (quality of evidence: B; strength of recommendation: strong).

### Is there an indication for surgical resection of metastases in patients with PC?

Patients with PC and distant metastases are considered unresectable, and palliative chemotherapy is the standard of care [8]. However, highly selected patients with resectable solitary hepatic or pulmonary metastases may potentially benefit from surgical resection when an R0 resection can be achieved by pancreatectomy and metastasectomy, when metastases remain stable or decrease in size with neoadjuvant chemotherapy and in patients without significant comorbidities and with good performance status [8, 40–42] (quality of evidence: C; strength of recommendation: weak).

### What should be the recommended annual minimum volume of patients per center and surgeon to obtain optimal surgical results in PC surgery?

An experienced high-volume pancreatic center is recommended for the surgical treatment of PC. Higher hospital volume is associated not only with reduced perioperative morbidity, mortality, length of hospital stay, and hospital costs but also with a higher chance of undergoing a radical resection, receiving adjuvant treatment, and longer survival [43–45]. Although consensus regarding the definition of high-volume centers and surgeons is needed, it is recommended that resections be performed at institutions that perform a large number (at least 15–20) of pancreatic resections annually [8]. Regarding the definition of high-volume surgeons, studies vary in the number of pancreaticoduodenectomies per year, ranging from 6 to > 20 pancreaticoduodenectomies/year [44] (quality of evidence: B; strength of recommendation: weak).

## Systemic treatment

### When is neoadjuvant treatment indicated?

Neoadjuvant treatment aims to increase overall survival by increasing the rate of R0 resection and early treatment of micrometastatic disease. For patients with resectable disease, neoadjuvant treatment cannot be recommended outside a clinical trial. Preoperative treatment for 3–4 months is the preferred approach in patients with borderline resectable disease [46, 47] (quality of evidence: B; strength of recommendation: moderate).

Afterwards, the patient should be under continuous evaluation by the multidisciplinary team. A lack of an objective radiological response should not be a criterion to rule out surgical resection. Patients with suspected disease progression by elevated CA 19.9 without radiological evidence of disease progression should be carefully evaluated, and PET scan and laparoscopy should be considered. Patients with documented metastatic progression are not candidates for surgery and should be managed as such [48].

### What is the neoadjuvant treatment indicated in patients with borderline tumors?

The chemotherapy treatments used should be those associated with a higher response rate in patients with metastatic disease (gemcitabine [GEM]/nab-paclitaxel, FOLFIRINOX) [6, 49]. Radiotherapy alone is not recommended and should be combined with either fluoropyrimidines or GEM [50, 51]. Patients who receive chemoradiation should wait four to eight weeks before attempting surgical resection (quality of evidence: B; strength of recommendation: moderate).

### Which neoadjuvant treatment is indicated in patients with unresectable tumors?

Whenever there are no data with regard to the most efficient regimen in this particular setting, current trends are to use either GEM/nab-paclitaxel or FOLFIRINOX based on the data available for patients with advanced disease [6, 52]. Chemotherapy is usually administered for 3 to 4 months followed by assessment of tumor response. Responding patients or patients with stable disease can continue chemotherapy, have a surgical resection or be treated with chemoradiation [51] (quality of evidence: B; strength of recommendation: moderate).

### When should adjuvant therapy be given after surgical resection of PC?

Even with R0 resection, the recurrence rate is very high in PC. Adjuvant treatment is recommended in patients who undergo an R0/R1 resection with a PT1-4/N0-1M0, with an ECOG performance status of 0–1 and proper nutritional status. As a result, adjuvant treatment is required in all patients with resected adenocarcinoma of the pancreas. It is often recommended that adjuvant treatment be initiated within the next 12 weeks after surgery in patients who do not have any serious postsurgical complication, active infection or signs or symptoms of recurrent disease [17] (quality of evidence: A; strength of recommendation: strong).

There is no consensus on adjuvant treatment in patients who have received neoadjuvant treatment. In general, adjuvant treatment in this population is still considered investigational. Generally, patients who have received neoadjuvant treatment should receive adjuvant treatment to complete a total of 6 months of treatment [53].

### What is the appropriate adjuvant treatment for patients with PC who underwent an R0 or R1 resection?

Currently, until the results of ongoing studies become available, the standard treatment is GEM in combination with capecitabine in the adjuvant setting in PC [54]. In patients not considered for combination treatment, the best option is single-agent GEM or the combination of 5-fluorouracil (5-FU) and folinic acid for a total of 6 months [55, 56] (quality of evidence: A; strength of recommendation: strong).

### What are the first-line treatment options for metastatic PC?

The management of patients with advanced PC is based on systemic chemotherapy. For patients who are able to receive chemotherapy without limitations, the current standard of care is either GEM/nab-paclitaxel or FOLFIRINOX [6, 7]. In the absence of randomized studies comparing these two regiments, neither one can be recommended. Patients with ECOG 2 can be treated with GEM alone [57] (quality of evidence: A; strength of recommendation: strong).

The response to treatment should be monitored every 8–12 weeks by a CT scan. The tumor marker CA 19.9 should be measured before treatment and every 4–8 weeks after treatment. Tumor progression in patients with rising CA 19.9 should be confirmed radiologically [17]. Patients who are not candidates for chemotherapy should receive palliative treatment.

### What is the treatment in patients with metastatic PC who progress after first-line chemotherapy?

Second-line treatment will be considered in selected patients with good performance status after progression to first-line treatment [58]. For patients who have been treated with a GEM-based regimen, the FOLFOX regimen demonstrated an improvement in survival compared to 5-FU in the CONKO-003 study [59]. However, these results with the addition of oxaliplatin to 5-FU were not confirmed in the PANCREOX trial [60]. Recently, the NAPOLI-1 study showed that NALIRI (liposomal formulation of irinotecan) in combination with 5-FU was better than 5-FU alone, with this combination being the best way to treat these patients [61]. For patients who have received 5-FU-based chemotherapy, there are very few data to base second-line choices on. In general, either GEM alone or a GEM combination is recommended [62] (quality of evidence: A; strength of recommendation: strong).

## Radiotherapy

### When is radiotherapy recommended in neoadjuvant treatment?

Initial neoadjuvant therapy is recommended for patients with borderline resectable disease based on small retrospective studies and meta-analyses. In this setting, the specific contribution of radiation therapy (RT) to neoadjuvant chemotherapy remains unclear. The goals of neoadjuvant RT are to decrease viable cells at the periphery of the tumor, thereby improving the chance of a negative margin [63, 64] (quality of evidence: B; strength of recommendation: moderate).

Neoadjuvant RT is usually administered concomitantly with chemotherapy after 2 or 3 cycles of induction chemotherapy if the patient remains free of distant metastases. Stereotactic body radiotherapy (SBRT) can be considered in the neoadjuvant setting instead of conventional fractionation RT [65], although no randomized trials comparing this approach with conventional RT have been completed.

### When is adjuvant radiation therapy indicated?

Chemoradiation has been used for resectable PC in the adjuvant setting based on its potential to decrease the likelihood of local recurrence and disease progression; however, the ESPAC-1 trial failed to show an advantage of the addition of postoperative radiation [66], with chemotherapy alone remaining the standard of care in the adjuvant setting.

In contrast, in the GERCOR phase II study, the rate of local recurrence was notably lower (11% vs 24%) for the group treated with chemoradiotherapy [67]. To definitively clarify the role of postoperative radiotherapy, the RTOG is conducting a trial with overall survival as the primary endpoint, which is estimated to be completed in 2020. In the meantime, some consideration of postoperative radiotherapy can be given to special cases of R1 disease (quality of evidence: B; strength of recommendation: moderate).

### What is the role of radiotherapy in the treatment of unresectable PC?

In patients with unresectable PC, most guidelines (NCCN, ASCO, ESMO) recommend an initial period of chemotherapy followed by either more chemotherapy or chemoradiotherapy [8, 68]. Chemoradiation is mainly used in selected patients who do not develop metastatic disease during initial chemotherapy [50, 51]. Chemoradiation can also be given as second-line therapy in patients with locally advanced unresectable disease if chemoradiation was not previously given and if the primary site is the sole site of progression. Finally, radiation alone can be used as palliative treatment for pain refractory to narcotic therapy (quality of evidence: B; strength of recommendation: moderate).

Chemoradiation can obtain higher loco-regional disease control in patients with unresectable PC compared with that of chemotherapy alone, although a benefit in overall survival has not been clearly demonstrated [69]. Chemoradiation can also increase the R0 resection rate in patients with unresectable disease who can finally be operated on [70].

### What are the most appropriate radiation doses and techniques to treat PC?

Pancreatic tumors are usually surrounded by multiple sensitive structures, such as the great vessels, the duodenum and the stomach. It is therefore important to use an advanced radiation technique capable of delivering a high dose of radiation to the tumor while minimizing toxicity to neighboring tissues. 3-D conformal RT (3D-CRT), intensity-modulated RT (IMRT), and SBRT can result in improved tumor coverage with decreased dose to adjacent organs at risk [51, 65, 71–73] (quality of evidence: B; strength of recommendation: strong).

For resectable/borderline/unresectable chemoradiation, 45–54 Gy in 1.8–2 Gy fractions is usually used. Additionally, 36 Gy in 2.4 fractions has been reported for preoperative chemoradiation [72]. SBRT uses hypofractionation, typically 3–7 fractions of 10–15 Gy. For adjuvant chemoradiation, the radiotherapy dose generally consists of 45–46 Gy in 1.8–2 Gy fractions to the tumor bed, surgical anastomoses and adjacent lymph node basins, potentially followed by an additional 5–9 Gy to the tumor bed [73].

## Palliative treatment

### For patients with unresectable/metastatic PC, what is the preferred method for the management of bile duct obstruction?

Endoscopic retrograde stenting is superior to surgical or percutaneous approaches because of a more favorable adverse event rate [74]. Self-expandable metal stents are superior to plastic stents in patients with a life expectancy of more than 3 months in terms of patency duration (approximately 8–12 vs 2–4 months), less therapeutic failure, less need for reintervention, lower cholangitis incidence and better patient quality of life [74–76]. Patency rates between covered and uncovered metal stents are not significantly different [77].

Percutaneous and endoscopic ultrasound (EUS)-guided biliary drainage are alternative methods if endoscopic biliary stent placement is unsuccessful or technically not feasible [23] (quality of evidence: A; strength of recommendation: strong).

### For patients with unresectable/metastatic PC, what are the recommended strategies for the management of gastric outlet obstruction?

Endoscopic duodenal stenting allows a quick resumption of oral intake, with a low complication rate and a short recovery period. However, the need for reintervention is higher after duodenal stenting compared with that of palliative surgery [78]. EUS-guided gastrojejunostomy has been developed as an effective and safe alternative to surgery [79] (quality of evidence: C; strength of recommendation: weak).

### For patients with unresectable/metastatic PC, what are the recommended strategies for the management of pain?

Optimal management of pain in PC should follow a multidisciplinary approach [80]. The main treatment options are chemotherapy, analgesics and interventional techniques. Chemotherapy may decrease pain by reducing tumor growth, local neural invasion and inflammation [81]. Current guidelines for analgesic therapy in PC follow the principles of the analgesic ladder provided by the World Health Organization [82]. EUS- or CT-guided celiac plexus neurolysis is the interventional technique of choice for pain in PC and should be evaluated mainly in patients with severe pain requiring a high dose of potent narcotics. It is associated with pain relief in 54–88% of cases, improved quality of life and decreased opioid consumption [83, 84] (quality of evidence: B; strength of recommendation: strong).

Palliative radiation therapy may also significantly alleviate pain due to local invasion of pancreatic cancer. A short course of external RT with or without concomitant chemotherapy is associated with the resolution of cancer-related pain in 35–65% of patients [85].

## Pathology

### Is it necessary to analyze intraoperative frozen sections of resection margins during pancreatic resections?

The pancreatic neck transection margin has been shown to be an important prognostic factor in PC and can be extended if tumor involvement is identified on intraoperative frozen sections to achieve a negative margin [86]. The common bile/hepatic duct transection margin should also be evaluated [87] (quality of evidence: A; strength of recommendation: strong).

### What is the best gross dissection protocol of the resection specimen of PC?

The use of a standardized pathology protocol based on axial slicing perpendicular to the long axis of the duodenum is recommended. The dissection technique results in 6–8 slices, allowing thorough examination of the tumor site and its relationship to the key anatomic structures (duodenum, common bile duct, peripancreatic soft tissue) and margins [88]. The examination of six distinct margins is recommended. The two transection margins are those of the pancreatic neck and the common bile duct. The four circumferential margins are the superior mesenteric vein margin, the superior mesenteric artery margin, the posterior margin, and the anterior surface of the pancreas [88, 89]. A careful sampling of the lymph nodes must be performed [88, 89] (quality of evidence: A; strength of recommendation: strong).

### When is a resection considered R0?

As a result of the infiltrating and often discontinuous pattern of PC growth, a minimum clearance of > 1 mm should be required to obtain a potentially curative resection [90]. R1 was defined when the distance of the tumor from the resection margin was ≤ 1 mm. The infiltration of the margin was further defined as “direct extension of the primary neoplasm” or lymph node metastasis or perineural/lymphatic/vascular tumor propagation ≤ 1 mm of the margin [91] (quality of evidence: A; strength of recommendation: strong).

### How many lymph nodes should be histologically examined to improve staging accuracy?

As in any other organs, the N-stage is one of the best prognosticators in PC. Studies have shown that the best prognostic value is achieved if 15 lymph nodes are examined, and patients with fewer lymph nodes evaluated following surgery may be understaged [90]. It should be noted that the total number of positive lymph nodes and the ratio of metastatic to examined lymph nodes (LNR) are powerful predictors of survival in patients with PC [90] (quality of evidence: A; strength of recommendation: strong).

### What pathological parameters should be evaluated in the assessment of the resection specimen with PC and after neoadjuvant therapy?

The histopathological assessment of resected specimens should include the parameters listed in Table 2. After neoadjuvant therapy, the College of American Pathologists (CAP) recommends a 4-tier scheme for tumor regression score for determining the treatment effect based on the amount of residual viable tumor cells [92, 93] (Table 3) (quality of evidence: A; strength of recommendation: strong).

**Table 2:** Pathological parameters evaluated in the assessment of the resection specimen with PC

-Type of specimen
-Maximum size of the tumor
-Histological type (WHO classification of exocrine pancreatic carcinomas) (Appendix A)
-Histological grading (Appendix B)
-Local invasion*
-Perineural, lymphatic and vascular vessel invasion
-Superior mesenteric vein or portal vein involvement
-Resection margins:
+Surgical transection margins:
•Pancreatic neck •Common bile duct
+ Circumferential resection margins:
•Superior mesenteric vein margin •Superior mesenteric artery margin •Posterior margin •Anterior surface of the pancreas
- Lymph node involvement
•Total number of nodes examined •Number of metastatic nodes
-UICC TNM staging (8^th^ edition)
-Completeness of excision (R category)

*Requires assessment of peripancreatic tissue invasion and involvement of the intrapancreatic common bile duct, duodenum and ampulla of Vater

**Table 3:** The CAP tumor regression grading system

Grade	Proportion of residual viable tumor
0	No viable cancer cells (complete histological response)
1	Single cells or rare small groups of cancer cells (nearly complete response)
2	Residual cancer with evident tumor regression, but more than single cells or rare small groups of cancer cells (partial response)
3	Extensive residual cancer with no evident tumor regression (poor or no response)

### What molecular-pathological studies should be performed in PC? What prognostic information do these studies provide?

Whole-exome sequencing reaffirmed known mutations in KRAS, TP53, CDKN2A, SMAD4, MLL3, TGFBR2, ARID1A and ATM [94]. More recent studies using next-generation sequencing techniques with whole-genome or exome sequencing have identified additional genetic alterations, including alterations in genes that play a central role in DNA repair. Germline and somatic mutations in the DNA damage repair genes BRCA2, BRCA1, PALPB2 and ATM were observed in 10% of samples, representing a class of patients for whom platinum-based chemotherapy and/or PARP inhibition may have therapeutic benefits [95].

One recently identified subtype within the genomic landscape of PC is the mismatch repair-deficient (MMR-D) tumor, which is present in < 1% of all PC patients and is typically associated with a germline mutation in MMR genes. MMR-D PC has a tendency to be associated with intraductal mucinous papillary neoplasm (IPMN) and a more favorable natural history. MMR-D PC has also been reported to have a medullary histology associated with the wild-type KRAS gene [96] (quality of evidence: B; strength of recommendation: moderate).

### What are the requirements of fine-needle aspiration (FNA) and fine-needle biopsy (FNB) for an adequate cytohistological evaluation of PC?

The diagnostic accuracy of EUS-FNA is reported to be over 90% in most studies when rapid on-site evaluation for cytopathology samples is employed. One or two passes usually allow the pathologist to evaluate the sample smears for diagnostic yield. Further passes may be made as needed to achieve diagnostic success [97] (quality of evidence: A; strength of recommendation: strong).

In theory, an FNB, or core-needle biopsy, contains a superior tissue sample with preserved cellular architecture compared to that from FNA. The randomized studies comparing FNA and core biopsy have produced different conclusions. Given the increased use of molecular studies on tissue samples required for gene-specific oncologic therapy, obtaining histologically sized specimens, rather than cytopathology, will be of importance in the future [97] (quality of evidence: B; strength of recommendation: moderate).

## Follow-up

### What is the recommended frequency of follow-up/surveillance after potentially curative treatment of PC?

MDCT is the primary imaging modality for monitoring following chemotherapy or surgery. Although MDCT scans may detect locally recurrent or metastatic disease, there is no evidence that regular follow-up after initial therapy with curative intent has any impact on the outcome [4, 17, 98]. We propose to follow-up patients with PC after surgical resection with measurement of tumor markers and a dynamic CT scan every 3–6 months for 2 years postoperatively and every 6–12 months subsequently, for at least 5 years postoperatively (quality of evidence: C; strength of recommendation: moderate). In unresectable PC, imaging intervals can be increased to every 6 months once stability is comfortably established [69], and in metastatic patients outside a clinical trial, to assess first response, a CT scan should be offered at 2 or 3 months after the initiation of therapy [98].

## Nutritional support, pancreatic exocrine insufficiency, and diabetes mellitus

### How and when do we assess nutritional status in patients with PC before surgery? If nutritional support is needed, by what means should it be administered?

Because malnourished patients suffer increased postoperative morbidity after duodenopancreatectomy, some nutrition assessment screening tools that track body mass index and the amount of weight loss are mandatory [99]. It is strongly recommended to start nutrition therapy early, as soon as a nutritional risk becomes apparent. Routine use of preoperative artificial nutrition is not warranted, but significantly malnourished patients should be optimized. A preoperative nutrition intervention plan includes dietary advice and oral nutritional supplements (ONS) if the dietary intake is less than 75% [99]. Parenteral nutrition should be used only if the enteral route is inaccessible [100] (quality of evidence: A; strength of recommendation: strong).

### What is the optimal nutritional support after surgery? What recommendations regarding diet and intake of nutrients can be given to patients with PC at discharge?

The ERAS guidelines [100] recommend a “normal diet”, as soon as possible, in patients at low risk of pancreatic fistula (first postoperative day [POD] drain fluid amylase < 350 IU/L) [101]. Patients should increase intake according to tolerance over 3–4 days. There was some heterogeneity in the planned schedule for the initiation of clear fluids (from 0–3 POD) and solid food/regular diet (from POD 3–5). ONS should be considered if oral intake is less than 75% [102]. An enteral feeding tube vs a catheter jejunostomy should be given only with specific indications. Parenteral nutrition should not be employed routinely [103]. After discharge, we recommended a normal diet, including 6 to 8 meals or snacks each day [102] (quality of evidence: B; strength of recommendation: strong).

### What is the recommended management for the treatment of diabetes mellitus and pancreatic exocrine insufficiency (PEI) before and after surgery for PC?

Type 3c diabetes occurs because of a variety of exocrine pancreatic diseases, including PC [104]. Metformin or insulin is used as a first-line therapy [105]. A high prevalence of PEI is observed in PC patients. Pancreatic enzyme replacement therapy (PERT) remains the mainstay of treatment. The initial dose is 75,000 Ph.U. of lipase/meal. Acid-suppressing therapy is frequently needed due to the reduced or abolished pancreatic bicarbonate secretion. Nutritional management by an experienced dietitian is essential [106] (quality of evidence: B; strength of recommendation: strong).

### What is the recommended nutritional support in patients with unresectable PC?

The nutritional status of patients with advanced cancer must be assessed since there is a clear benefit of nutritional supplementation on survival time, performance status, and QoL [107]. It is important that vitamin D and PEI be treated as well as other concomitant symptoms that affect appetite and food intake, such as mechanical or functional gastrointestinal disorders, depression and fatigue [108]. Specific nutrients, such as N3-fatty acids, l-carnitine, antioxidants, branched-chain amino acids and lactoferrin, can be administered to fight cachexia, but the overall results remain inconclusive [107, 108]. For patients with cancer who are nearing the end of life, nutrition is tailored to the patient’s symptomatic needs and is primarily intended to support comfort and QoL [109] (quality of evidence: B; strength of recommendation: strong).

### What is the recommended management for the treatment of diabetes mellitus and PEI in patients with unresectable PC?

PEI is very frequent (> 90% when the tumor is located in the head of the pancreas), and it has been associated with higher mortality in patients with unresectable PC [110]. Pancreatic enzyme replacement therapy improves survival in these patients [111, 112]. The recommended starting dose is 75,000 Ph.U./meal. The addition of a PPI is frequently needed. The diet should not be low in fat to achieve a better effect [113]. The elastase-1 stool test has been shown to be a simple, noninvasive, low-cost technique with an acceptable correlation with secretory tests [113].

The presence of diabetes has been associated with higher mortality in patients with unresectable PC [114]. High-dose glucocorticosteroids can induce or exacerbate diabetes. Careful monitoring of plasma glucose levels 2 h after lunch is widely recommended. The limited literature on this topic recommends maintaining blood glucose levels to avoid hypoglycemia and reduce symptoms of hyperglycemia [115]. Insulin is considered the preferred agent because of its efficacy, flexibility, and safety [115] (quality of evidence: B; strength of recommendation: strong).

## Electronic supplementary material

Below is the link to the electronic supplementary material.

Supplementary file1 (DOCX 15 kb)
