# Supplementary material for: Multidisciplinary consensus statement on the clinical management of patients with pancreatic cancer
Source: Clin Transl Oncol. 2020 Apr 21;22(11):1963–75. doi: 10.1007/s12094-020-02350-6 (PMC7505812; doi:10.1007/s12094-020-02350-6)
Supplement: Supplementary file 1 — Supplementary file1 (DOCX 15 kb) [file 12094_2020_2350_MOESM1_ESM.docx]

**Supplementary Appendix**

**Appendix A**. WHO classification of malignant exocrine carcinomas and SNOMED “M” codes

| **Morphological codes** | **SNOMED code** |
| --- | --- |
| Ductal adenocarcinoma | M8500/3 |
| Adenosquamous carcinoma | M8560/3 |
| Colloid carcinoma (mucinous noncystic carcinoma) | M8480/3 |
| Hepatoid carcinoma | M8576/3 |
| Medullary carcinoma | M8510/3 |
| Signet-ring carcinoma | M8490/3 |
| Undifferentiated (anaplastic or sarcomatoid) carcinoma | M8020/3 |
| Undifferentiated carcinoma with osteoclast-like giant cells | M8035/3 |
| Acinar cell carcinoma | M8550/3 |
| Acinar cell cystadenocarcinoma | M8551/3 |
| Intraductal papillary mucinous neoplasm with an associated invasive carcinoma | M8453/3 |
| Mixed acinar-ductal carcinoma | M8552/3 |
| Mixed acinar-neuroendocrine carcinoma | M8154/3 |
| Mucinous cystic neoplasm with an associated invasive carcinoma | M8470/3 |

**Appendix B**. Histological grading of pancreatic ductal adenocarcinoma

| **Tumor grade/**  **differentiation** | **Duct structures** | **Nuclei** | **Mitotic figures per 10 high-power fields** | **Mucin production** |
| --- | --- | --- | --- | --- |
| Grade 1, well differentiated | Well-formed duct-like structures and tubular glands | Little polymorphism, polar arrangement | ≤ 5 | Intensive |
| Grade 2, moderately differentiated | Some well-formed duct-like structures and tubular glands | Moderate polymorphism | 6-10 | Irregular |
| Grade 3, poorly differentiated | Abortive mucoepidermoid and pleomorphic structures | Marked polymorphism and increased size | > 10 | Abortive |

Based on Klöppel G, Solcia E, Longnecker DS, Capella C, Sobin LH. Histological Typing of Tumours of the Exocrine Pancreas (2nd edn), WHO International Histological Classification of Tumours. Springer-Verlag: Berlin, 1996..
